# Comparison of the Modified TyG Indices and Other Parameters to Predict Non-Alcoholic Fatty Liver Disease in Youth

**DOI:** 10.3390/biology11050685

**Published:** 2022-04-29

**Authors:** Kyungchul Song, Hae Won Lee, Han Saem Choi, Goeun Park, Hye Sun Lee, Su Jin Kim, Myeongseob Lee, Junghwan Suh, Ahreum Kwon, Ho-Seong Kim, Hyun Wook Chae

**Affiliations:** 1 Department of Pediatrics, Severance Children’s Hospital, Endocrine Research Institute, Yonsei University College of Medicine, Seoul 03722, Korea; endosong@yuhs.ac (K.S.); hione@yuhs.ac (H.W.L.); nounours89@yuhs.ac (S.J.K.); mslee8791@yuhs.ac (M.L.); suh30507@yuhs.ac (J.S.); armea@yuhs.ac (A.K.); kimho@yuhs.ac (H.-S.K.); 2 Department of Pediatrics, International St. Mary’s Hospital, Catholic Kwandong University, Incheon 22711, Korea; hansaem6890@gmail.com; 3 Biostatistics Collaboration Unit, Yonsei University College of Medicine, Seoul 03722, Korea; gogoeun@yuhs.ac (G.P.); hslee1@yuhs.ac (H.S.L.)

**Keywords:** triglycerides, glucose, biomarkers, non-alcoholic fatty liver disease, child, adolescent

## Abstract

**Simple Summary:**

Non-alcoholic fatty liver disease (NAFLD) is associated with cardio-metabolic risk factors, including obesity, dyslipidemia, insulin resistance, and hepatic cirrhosis. The increasing prevalence of NAFLD among youths has become a public health concern. However, studies about reliable markers for assessing NAFLD in youths are limited. Thus, we investigated the markers including the triglycerides-glucose (TyG) index, modified TyG indices, hepatic steatosis index (HSI), aspartate transaminase-to-platelet ratio index (APRI), and modified APRIs for the prediction of NAFLD. This study demonstrated that the modified TyG indices, APRI-body mass index standard deviation score, and HSI are strongly associated with NAFLD in children and adolescents. Thus, these markers may be useful for identifying youths who require hepatic ultrasonography and early treatment.

**Abstract:**

We investigated the modified triglycerides-glucose (TyG) indices and other markers for non-alcoholic fatty liver disease (NAFLD) in 225 participants aged 10–19 years, and the participants were divided into subgroups according to their NAFLD grade. We performed logistic regression analysis and calculated the odds ratios (ORs) with 95% confidence intervals (CIs) of tertiles 2 and 3 for each parameter, with those of tertile 1 as a reference. The area under the receiver operating characteristic (ROC) curve was calculated to compare the parameters for identifying NAFLD. TyG and modified indices, aspartate transaminase-to-platelet ratio index (APRI)-body mass index (BMI), APRI-BMI standard deviation score (SDS), APRI waist-to-hip ratio, fibrosis-4 index (FIB)-4, and hepatic steatosis index (HSI) were higher in participants with NAFLD than in those without NAFLD. The ORs and 95% CIs for NAFLD progressively increased across tertiles of each parameter. TyG and modified TyG indices, FIB-4, HSI, and modified APRIs, except APRI waist-to-height ratio, predicted NAFLD significantly through ROC curves. Modified TyG indices, APRI-BMI SDS, and HSI were superior to the other markers for NAFLD prediction. Modified TyG indices, APRI-BMI SDS, and HSI appear to be useful for assessing NAFLD in youths.

## 1. Introduction

Non-alcoholic fatty liver disease (NAFLD), a chronic liver disease caused by excessive fat accumulation in the liver, falls under a spectrum of progressive liver diseases that include simple steatosis, non-alcoholic steatohepatitis, and hepatic fibrosis [1,2]. NAFLD is associated with cardio-metabolic risk factors including obesity, dyslipidemia, insulin resistance (IR), and hepatic cirrhosis [2,3]. Moreover, the prevalence of NAFLD among children and adolescents increased from 3.9% in 1988–1994 to 10.7% in 2007–2010 in the United States. In Korea, the prevalence increased from 8.17% in 2009 to 12.05% in 2018 [1,4]. Thus, monitoring NAFLD in children and adolescents is a crucial task for pediatricians.

NAFLD is usually asymptomatic and often reversible with early treatment [5]. Thus, early monitoring of NAFLD is important to prevent serious complications. Various parameters, including the aspartate transaminase (AST)-to-platelet ratio index (APRI) and fibrosis-4 index (FIB-4), which are derived from AST, alanine aminotransferase (ALT), and/or platelets, have been used to assess histologic change in NAFLD in previous studies on adults [6,7]. A systematic review of NAFLD guidelines suggested that fibrosis scores, including FIB-4, are acceptable predictors for identifying patients with a risk of hepatic fibrosis [8]. Moreover, the association between these parameters and hepatic steatosis has been reported in previous studies [9,10]. Rihamonti et al. [10] reported that APRI was a useful predictive marker for NAFLD in youth with obesity. A meta-analysis reported that FIB-4 predicted non-alcoholic steatohepatitis and liver fibrosis more precisely than other parameters, including APRI [11]. In addition, the triglyceride-glucose (TyG) index, which is a parameter derived as the product of levels of fasting glucose and triglycerides (TG), has been suggested to be a useful predictive marker for NAFLD, because of the association of NAFLD with IR and dyslipidemia [12,13].

Since NAFLD is strongly related with obesity, studies have suggested including obesity indices such as the body mass index (BMI), waist circumference (WC), and waist-to-height ratio (WHtR) for assessing NAFLD [6,14,15]. Modified TyG indices, the TyG index combined with obesity indices, have been suggested as better predictive markers than TyG alone in adults [14,15]. Additionally, the hepatic steatosis index (HSI), a marker derived from the sum of the ALT/AST ratio and BMI, has been suggested as a useful screening tool for NAFLD [6,16]. However, investigations comparing TyG and modified TyG indices and other NAFLD-related parameters are limited in the young population.

Therefore, this study primarily aimed to investigate the association of TyG and modified TyG indices and NAFLD-related parameters among children and adolescents with NAFLD using a health checkup program database. Our objectives were as follows: (1) to compare TyG and modified TyG indices and other NAFLD-related parameters as predictive markers for NAFLD, and (2) to determine valid cut-off values of TyG index, modified TyG indices, and other NAFLD-related parameters for predicting NAFLD.

## 2. Materials and Methods

### 2.1. Study Participants

**Figure 1:**
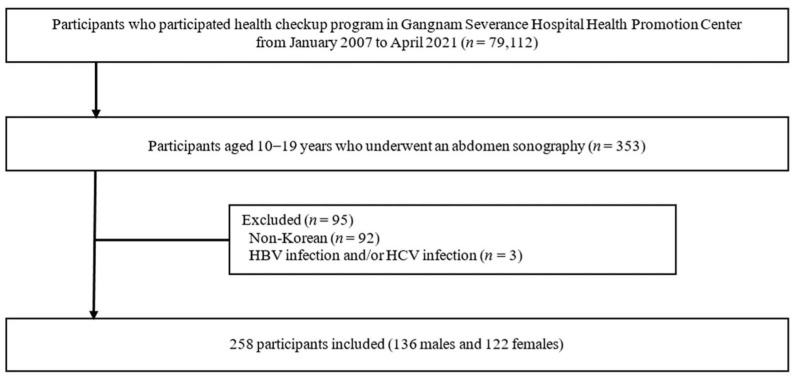
Design and flowchart of the study population. HBV, hepatitis B virus; HCV, hepatitis C virus.

We conducted a retrospective cross-sectional analysis of data from 258 children and adolescents aged 10–19 years who participated in a health checkup program at the Gangnam Severance Hospital Health Promotion Center in Korea from January 2007 to April 2021. Figure 1 illustrates the study design and workflow. None of the participants had diabetes. In addition, none of the participants reported alcohol use on subject-recorded questionnaires.

### 2.2. Anthropometric Measurements

Height was measured to the nearest 0.1 cm, and body weight was measured by an electronic weighting scale to the nearest 0.01 kg, with the participants wearing a light gown without shoes for the health examination. BMI was calculated as weight in kilograms divided by the square of height in meters (kg/m^2^). Height, weight, and BMI were presented as standard deviation score (SDS) values based on the 2017 Korean National Growth Charts [17]. Children were classified as normal (<85th percentile), overweight (85th–95th percentile), or obese (≥95th percentile) according to their BMI. Waist and hip circumferences were measured at the midpoint between the lower margin of the least palpable rib and the top of the iliac crest in the horizontal plane for the waist, and around the widest portion of the buttocks for the hip, respectively, by a trained nurse. The WHtR was calculated as WC (cm)/height (cm), and the waist-to-hip ratio (WHR) was calculated as WC (cm)/hip (cm). Central obesity was defined as a WC > 90th percentile [18].

### 2.3. Laboratory Analyses

The blood samples were collected from all participants after 8 h of fasting, immediately centrifuged, and stored at −70 °C before analysis. The levels of fasting plasma glucose, total cholesterol (TC), high-density lipoprotein cholesterol (HDL-C), TG, AST, and ALT were assessed using enzymatic procedures, with an automated chemistry analyzer (Hitachi 7600–120, Hitachi, Tokyo, Japan). Low-density lipoprotein cholesterol (LDL-C) levels were calculated using the Friedewald formula (LDL-C = TC − [HDL-C + (TG/5)]) [19], and non-HDL-C levels were calculated as TC − HDL-C [20]. The levels of hepatitis B surface antigen and anti-hepatitis C virus antibodies were measured using the Roche E-170 device (Roche Diagnostics, Mannheim, Germany).

The TyG index and modified TyG indices were calculated using the following formulae [2,21]:

TyG index = Ln [TG (mg/dL) × fasting glucose (mg/dL)/2]

TyG-BMI = TyG index × BMI

TyG-BMI SDS = TyG index × BMI SDS

TyG-WC = TyG index × WC

TyG-WHR = TyG index × WHR

TyG-WHtR = TyG index × WHtR

The APRI and modified APRIs were calculated as follows [22]:

APRI = AST level/upper level of normal AST/platelet counts (10^9^/L) × 100

APRI-BMI = APRI × BMI

APRI-BMI SDS = APRI × BMI SDS

APRI-WC = APRI × WC

APRI-WHR = APRI × WHR

APRI-WHtR = APRI × WHtR

FIB-4 was calculated as age × AST level/platelet count × √ALT [23]. The HSI was calculated as 8 × ALT/AST ratio + BMI (+2, if diabetes; +2, if female) [6].

### 2.4. Ultrasonographic Analyses

Fatty liver disease was diagnosed based on the findings of an abdominal ultrasonography scan performed using a 3.5 MHz transducer (HDI 5000, Philips, Bothell, WA, USA). One of three experienced radiologists performed the ultrasonographic examination without information about the participants. The participants were classified into four groups based on the existence and severity of NAFLD according to the level of hepatic tissue echogenicity, the discrepancy between the liver and right kidney, and the visibility of the vascular structures [24]. Grades 1–3 of hepatic fat accumulation were considered as NAFLD, and grade 0 was considered as normal.

### 2.5. Statistical Analyses

All continuous variables are presented as the mean ± standard deviation, and categorical variables are expressed as numbers (percentages). The independent t-test and analysis of variance were performed to compare the continuous variables. The chi-square test was performed to compare the categorical variables with percentages after dividing participants into subgroups according to the presence of NAFLD or the NAFLD grade. Logistic regression analyses were performed to elucidate the relationship between NAFLD as the dependent variable and various markers. Odds ratios (ORs) and 95% confidence intervals (CIs) of tertiles 2 and 3 for each parameter were calculated and compared with tertile 1 as a reference. The area under the curve (AUC) of the receiver operating characteristic (ROC) curve was calculated to compare the diagnostic strengths of these parameters in identifying NAFLD. Sensitivity and specificity were calculated as the markers’ optimal cut-off values based on the Youden index. The bootstrap method was used to perform pairwise comparisons between AUCs for the parameters. The data were analyzed using SAS, version 9.4 (SAS Inc., Cary, NC, USA), and R, version 4.1.0 (The R Foundation for Statistical Computing, Vienna, Austria; http://www.R-project.org, accessed on 2 June 2021). Statistical significance was determined as *p* < 0.05. 

## 3. Results

### 3.1. Characteristics and Parameters of the Participants According to the NAFLD Grade

Table 1 shows the baseline characteristics of participants according to the presence of NAFLD. Overall, the proportion of participants with NAFLD was 11.24%, and the proportions of those with NAFLD grades 0, 1, 2, and 3 were 87.98%, 6.59%, 2.71%, and 2.71%, respectively. The BMI, BMI SDS, WC, WHR, and WHtR and the proportion of participants with obesity and central obesity were higher among participants with NAFLD than among those without NAFLD. AST, ALT, gamma-glutamyl transferase, TG, and non-HDL-C levels were higher in participants with NAFLD than in those without NAFLD, while the HDL-C level was lower in participants with NAFLD than in those without NAFLD (Table S1). TC/HDL-C, TG/HDL-C, LDL-C/HDL-C, and non-HDL-C/HDL-C values were higher in participants with NAFLD than in those without NAFLD. Further, the TyG index and all modified TyG indices were higher in participants with NAFLD than in those without NAFLD. APRI-BMI, APRI-BMI SDS, APRI-WHR, FIB-4, and HSI were higher in participants with NAFLD than in those without NAFLD.

**Figure 2:**
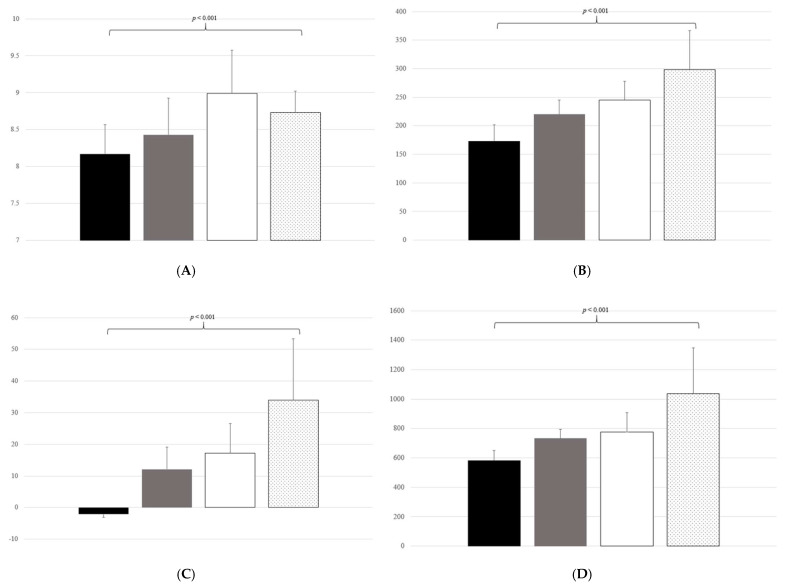

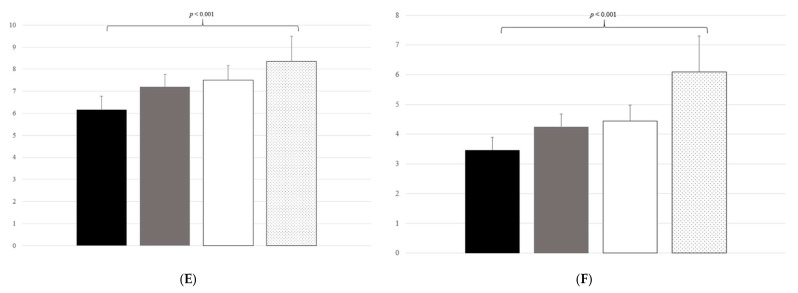
TyG and modified TyG indices according to the NAFLD grade. The black bar is the NAFLD grade group, the gray bar is the NAFLD grade 1 group, the white bar is the NAFLD grade 2 group, and the bar with the dotted pattern is the NAFLD grade 3 group. The number on the bar is the *p*-value of the analysis of variance. (**A**) TyG index according to the NAFLD grade. (**B**) TyG-BMI according to the NAFLD grade. (**C**) TyG-BMI SDS according to the NAFLD grade. (**D**) TyG-WC according to the NAFLD grade. (**E**) TyG-WHR according to the NAFLD grade. (**F**) TyG-WHtR according to the NAFLD grade. TyG, triglyceride glucose index; NAFLD, non-alcoholic fatty liver disease; BMI, body mass index; SDS, standard deviation score; WC, waist circumference; WHR, waist-to-hip ratio; WHtR, waist-to-height ratio.

Figure 2A–F show the TyG index and modified TyG indices among the subgroups classified by the NAFLD grade. All modified TyG indices increased with an increase in the NAFLD grade (all, *p* < 0.001). The TyG index was significantly higher in participants with grade 1 than in those with grade 0, and it was significantly higher in participants with grade 2 than in those with grade 1; however, the TyG index was not significantly different between participants with grade 2 and those with grade 3. In addition, APRI-BMI SDS and HSI increased with an increase in the NAFLD grade (all, *p* < 0.001) (Figure S1A–H).

### 3.2. ORs of the Parameters for Predicting NAFLD

The TC/HDL-C, TG/HDL-C, LDL-C/HDL-C, non-HDL-C/HDL-C, TyG index, all modified TyG indices, APRI-BMI, APRI-BMI SDS, APRI-WHR, FIB-4, and HSI showed significantly higher ORs and 95% CIs for tertile 3 as compared to that in tertile 1 (TyG-BMI, TyG-BMI SDS, and APRI-BMI SDS, *p* = 0.002; TyG-WC and TyG-WHtR, *p* = 0.026; TyG-WHR, *p* = 0.003; APRI-WHR, *p* = 0.004; FIB-4, *p* = 0.001; HSI, *p* = 0.003; others, *p* < 0.001) (Table 2). TyG-BMI, TyG-BMI SDS, and APRI-BMI SDS had the highest ORs and 95% CIs for NAFLD (OR, 84.284), which were followed by HSI (OR, 79.035) and TyG-WHR (OR, 73.974). Overall, the modified TyG indices had higher ORs and 95% CIs than the TyG index, and modified APRIs had higher ORs and 95% CIs than the APRI. The HSI had higher ORs and 95% CIs than the TyG index, APRI, and FIB-4.

### 3.3. Cut-Off Values and AUC of the Parameters for Predicting NAFLD

**Figure 3:**
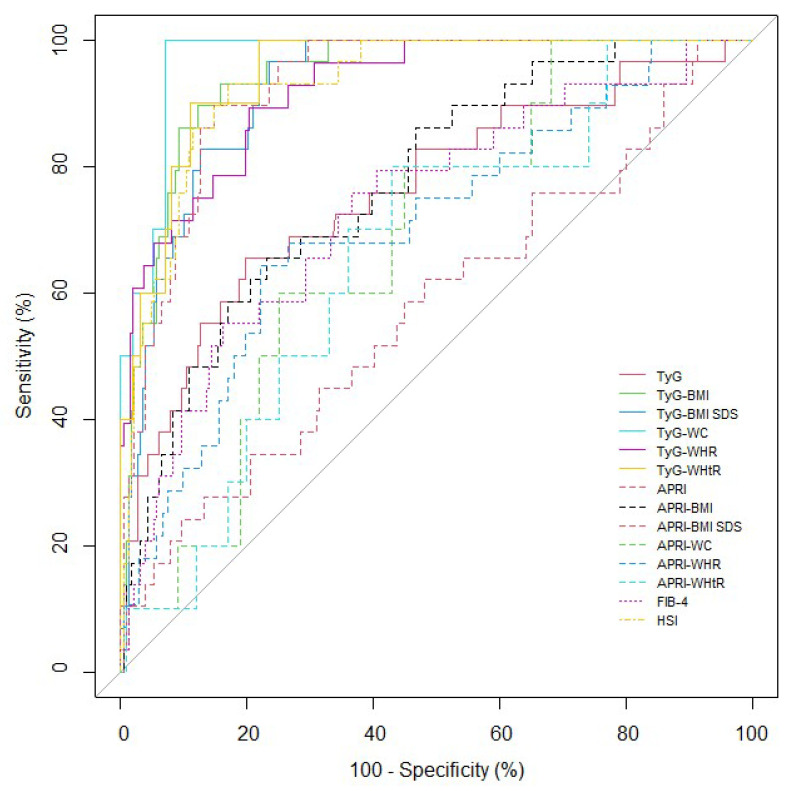
ROC for each variable to predict NAFLD in the participants. NAFLD, non-alcoholic fatty liver disease. TyG: triglyceride glucose index; BMI: body mass index; SDS: standard deviation score; WC: waist circumference; WHR: waist-to-hip ratio; WHtR: waist-to-height ratio; APRI: aspartate aminotransferase to platelet index; FIB-4: Fibrosis-4 index; HSI: hepatic steatosis index.

The results of ROC curve analyses and AUCs with corresponding 95% CIs for the parameters to predict NAFLD are shown in Table 3 and Figure 3. AUC values ranged from 0.575 to 0.972 for all the participants. All the parameters, except APRI and APRI-WHtR, could significantly predict NAFLD (APRI-WC, *p* = 0.019; others, *p* < 0.001). TyG-WC exhibited the largest AUC for detecting NAFLD at 0.972 (*p* < 0.001). All the modified TyG indices exhibited significantly higher AUC values and 95% CIs than the TyG index and FIB-4 (Comparison between TyG index and TyG-BMI SDS, *p* = 0.003; others, *p* < 0.001) (Table S2). Among the modified APRIs, APRI-BMI, APRI-BMI SDS, and APRI-WHR demonstrated significantly higher AUC values and 95% CIs than APRI (all, *p* < 0.001). In addition, APRI-BMI SDS demonstrated significantly higher AUC values and 95% CIs than the TyG index and APRI-BMI (TyG, *p* = 0.002; APRI-BMI, *p* < 0.001). HSI demonstrated significantly higher AUC values and 95% CIs than the TyG index, APRI, APRI-BMI, APRI-WC, APRI-WHR, APRI-WHtR, and FIB-4 (APRI-WC and APRI-WHtR, *p* = 0.002; others, *p* < 0.001).

## 4. Discussion

This study demonstrated that modified TyG indices, APRI-BMI SDS, and HSI could be used as powerful predictors for NAFLD in youths. The ORs and 95% CIs for NAFLD progressively increased across the tertiles for each parameter. Further, the modified TyG indices, APRI-BMI SDS, and HSI presented higher ORs and 95% CIs for predicting NAFLD compared to those of the TyG index, FIB-4, APRI, and other modified APRIs. In the ROC curve analysis, the TyG index and modified TyG indices, FIB-4, HSI, and modified APRIs (except APRI-WHtR) could significantly predict NAFLD. Among the indices, TyG-WC was the most powerful predictor. Our findings suggest that the novel parameters, TyG-WHR and the modified APRIs, are useful predictors for NAFLD. In addition, modified TyG indices, APRI-BMI SDS, and HSI, which combined obesity parameters with the levels of liver enzymes or glucose and TG, had a better diagnostic value than using the conventional TyG index and APRI for predicting NAFLD in youths.

In our study, the AUC range of modified TyG indices for NAFLD detection was high, at 0.923–0.972. This range was higher than that reported in our previous study, wherein NAFLD was defined by ALT elevation [2]. Although the ALT level is recommended as a screening tool for NAFLD in children who present with risk factors for NAFLD, some children with NAFLD have a normal ALT level, and a high ALT level is also shown in other liver diseases [5,25]. Moreover, ultrasonography can exclude other liver diseases, and assess the degree of fatty infiltration by the degree of echogenecity [5,26]. The AUC for ultrasonographic detection of moderate-to-severe hepatic steatosis was high, at 0.87, in a prospective study [27]. A cross-sectional study reported a positive relationship between the TyG index and hepatic steatosis diagnosed using ultrasonography in overweight and obese youths [28]. Our study strengthened the relationship between modified TyG indices and NAFLD in youths with a normal BMI by using ultrasonography to diagnose NAFLD.

In our study, the TyG index was superior to APRI and FIB-4, and modified TyG indices were superior to modified APRIs. Insulin promotes the maturation of adipocytes and the uptake of fatty acids from circulating lipoprotein [2,29]. Hypertriglyceridemia promotes the transport of free fatty acids to the liver and increases hepatic glucose output [29,30]. Thus, the TyG index is closely associated with IR and NAFLD [2,21]. APRI and FIB-4 are derived from liver markers including AST, ALT, and/or platelet values. The superiority of the TyG index, compared to that of APRI and FIB-4 in our study, demonstrates the strong association of NAFLD with IR and hypertriglyceridemia. Zhang et al. [31] reported that the AUC of the TyG index for detecting NAFLD was higher than that of the ALT level in adults. Further, Simental-Mendía et al. [28] reported that the OR of the TyG index for detecting fatty liver was higher than that for AST and ALT levels in youths. Ye et al. [32] reported that the TyG index was a useful biomarker for NAFLD and could be more powerful when combined with ALT in children.

In our study, modified TyG indices including new parameters, TyG-WHR and HSI, which were combined with obesity indices, were superior to other markers, and modified APRIs were superior to the APRI. Based on the association of obesity with NAFLD and IR, modified TyG indices have been suggested as better markers than the TyG index alone for detecting NAFLD [14,15,33]. A population-based study reported that TyG-BMI was superior to the TyG index for predicting NAFLD [15]. Kim et al. [14] reported that TyG-BMI and TyG-WC were superior to the TyG index and homeostatic model assessment for IR in predicting NAFLD. Sheng et al. [34] reported that TyG-BMI was the best predictor among 15 NAFLD-related parameters in young males. Further, our previous studies demonstrated the superiority of modified TyG indices for predicting NAFLD and IR compared to the TyG index alone in youths [2,21].

We proposed novel parameters such as modified APRIs that could significantly predict NAFLD, including APRI-BMI, APRI-BMI SDS, APRI-WC, and APRI-WC. Among the modified APRIs, APRI-BMI SDS was the most powerful predictor for detecting NAFLD. Since obesity is defined as BMI > 95th percentile according to the sex and age of children and adolescents, BMI SDS is used more commonly than BMI [33]. The North American Society of Pediatric Gastroenterology, Hepatology, and Nutrition guidelines suggest screening for NAFLD in children with a BMI ≥ 95th percentile or BMI ≥ 85th percentile with risk factors [5]. Thus, we proposed a novel predictor, TyG-BMI SDS, which was superior to the TyG index in predicting IR and NAFLD. Further, it was the most powerful predictive marker among the modified TyG indices for IR in youths [2,21]. There was no significant difference between the predicted values of TyG-BMI and TyG-BMI SDS in our study, which could be due to a stronger relationship of TyG with NAFLD compared to that with APRI.

In our study, HSI was superior to APRI and FIB-4. HSI was proportional to the ALT level, APRI to the AST level, and FIB-4 to AST and √ALT values [6,22,23]. The ALT level has adequate sensitivity for detecting NAFLD, whereas the AST level has not been suggested as an independent screening tool for NAFLD in children [5,35]. Further, the AST level increases with the progression of hepatic fibrosis in the context of ALT elevation, although an elevated ALT level is not correlated with the severity of NAFLD histology [5,22,36]. Based on the evidence, APRI and FIB-4 have been suggested as markers for hepatic fibrosis, and HSI has been suggested as a marker for hepatic steatosis [6,22,23,37]. Yang et al. [3] reported that the APRI and FIB-4 might be useful for predicting hepatic fibrosis in children with NAFLD. Ferraioli et al. [37] reported that HSI is positively associated with the presence of hepatic steatosis in children. Shi et al. [38] reported that HSI was superior to the TyG index for NAFLD prediction in children.

The present study had some limitations. First, this study included a low number of participants and was limited to a single center in South Korea. Further studies are needed to validate the predictability of novel parameters using the heterogeneous composition of anthropometric data from a larger and regionally diversified population. Second, factors associated with NAFLD such as nutrition, exercise, and puberty were not considered in this study. Third, we were unable to consider lean and fat body masses. Fourth, a liver biopsy was not performed, which is the gold standard technique for identifying hepatic steatosis. However, we demonstrated the association of NAFLD with various NAFLD-related markers using ultrasonography. In addition, we proposed new predictors, TyG-WHR and modified APRIs, for detecting NAFLD in youths. 

## 5. Conclusions

Our findings suggest novel parameters, including TyG-WHR and modified APRIs, as useful markers for NAFLD prediction in youth. Furthermore, modified TyG indices, APRI-BMI SDS, and HSI were found to be strongly associated with NAFLD in children and adolescents. Moreover, modified TyG-indices, APRI-BMI SDS, and HSI have better predictability than the TyG index, FIB-4, APRI, and other modified APRIs. The modified TyG indices, APRI-BMI SDS, and HSI are easily available and economical markers for NAFLD. Thus, these markers may be useful for identifying youths who require hepatic ultrasonography and early treatment.

## Figures and Tables

**Table 1:** Characteristics of participants according to NAFLD.

Characteristics	Total (*n* = 258)	Normal (*n* = 229)	NAFLD (*n* = 29)	*p*
Age, years	18.02 ± 1.41	18.00 ± 1.42	18.14 ± 1.33	0.621
Male, *n* (%)	136 (52.71)	113 (49.34)	23 (79.31)	0.002
Height SDS	0.22 ± 1.08	0.19 ± 1.06	0.45 ± 1.24	0.221
Weight SDS	0.13 ± 1.4	−0.11 ± 1.21	1.99 ± 1.38	<0.001
BMI, kg/m^2^	22.03 ± 4.12	21.24 ± 3.19	28.25 ± 5.27	<0.001
BMI SDS	0.01 ± 1.48	−0.25 ± 1.24	2.12 ± 1.56	<0.001
BMI grade, *n* (%) Normal	200 (77.52)	195 (85.15)	5 (17.24)	<0.001
Overweight	27 (10.47)	21 (9.17)	6 (20.69)	
Obesity	31 (12.02)	13 (5.68)	18 (62.07)	
WC, cm	73.34 ± 10.8	71.29 ± 7.29	93.8 ± 17.78	0.003
Central obesity, *n* (%)	10 (9.09)	5 (5)	5 (50)	<0.001
WHR	0.77 ± 0.07	0.76 ± 0.06	0.87 ± 0.09	<0.001
WHtR	0.44 ± 0.06	0.43 ± 0.05	0.54 ± 0.1	0.004
Glucose, mg/dL	90 ± 7.17	89.84 ± 7.07	91.28 ± 7.95	0.310
AST, IU/L	20.79 ± 9.38	20.25 ± 9.18	25.1 ± 9.98	0.008
ALT, IU/L	18.78 ± 16.98	16.21 ± 12.02	39.07 ± 31.46	<0.001
TC/HDL-C	3.17 ± 0.72	3.07 ± 0.63	3.97 ± 0.88	<0.001
TG/HDL-C	1.87 ± 1.76	1.68 ± 1.54	3.37 ± 2.54	0.001
LDL-C/HDL-C	1.93 ± 0.6	1.85 ± 0.55	2.56 ± 0.63	<0.001
Non-HDL-C/HDL-C	2.17 ± 0.72	2.07 ± 0.63	2.97 ± 0.88	<0.001
TyG	8.22 ± 0.44	8.17 ± 0.4	8.63 ± 0.54	<0.001
TyG-BMI	181.76 ± 39.15	173.88 ± 29.45	243.96 ± 49.92	<0.001
TyG-BMI SDS	0.32 ± 12.5	−1.97 ± 10.26	18.36 ± 13.97	<0.001
TyG-WC	603.33 ± 105.26	582.8 ± 69.55	808.65 ± 171.61	0.002
TyG-WHR	6.34 ± 0.79	6.18 ± 0.61	7.57 ± 0.89	<0.001
TyG-WHtR	3.59 ± 0.59	3.48 ± 0.42	4.69 ± 0.92	0.002
APRI	0.33 ± 0.15	0.32 ± 0.14	0.37 ± 0.18	0.077
APRI-BMI	7.16 ± 3.41	6.76 ± 2.98	10.33 ± 4.76	<0.001
APRI-BMI SDS	0.00 ± 0.54	−0.10 ± 0.46	0.74 ± 0.56	<0.001
APRI-WC	25.57 ± 10.23	25.03 ± 9.63	30.95 ± 14.55	0.237
APRI-WHR	0.25 ± 0.11	0.24 ± 0.09	0.33 ± 0.15	0.005
APRI-WHtR	0.15 ± 0.06	0.15 ± 0.06	0.18 ± 0.08	0.127
FIB-4	6.39 ± 5.93	5.8 ± 4.98	11.1 ± 9.74	0.007
HSI	29.8 ± 6.28	28.53 ± 4.65	39.89 ± 8.15	<0.001
NAFLD grade				<0.001
Grade 0, *n* (%)	227 (87.98)	227 (100.00)	0 (0.00)	
Grade 1, *n* (%)	17 (6.59)	0 (0.00)	17 (58.62)	
Grade 2, *n* (%)	7 (2.71)	0 (0.00)	7 (24.13)	
Grade 3, *n* (%)	7 (2.71)	0 (0.00)	7 (24.13)	

Continuous variables are presented as mean ± standard deviation and categorical variables as numbers (percentages). *p*-value is assessed using an independent *t*-test for continuous variables and the chi-square test for categorical variables. Central obesity was defined as a WC > 90th percentile. NAFLD: Non-alcoholic fatty liver disease; SDS: standard deviation score; BMI: body mass index; WC: waist circumference; WHR: waist-to-hip ratio; WHtR: waist-to-height ratio; AST: aspartate aminotransferase; ALT: alanine aminotransferase; TC: total cholesterol; TG: triglycerides; HDL-C: high-density lipoprotein cholesterol; LDL-C: low-density lipoprotein cholesterol; TyG: triglyceride glucose index; APRI: AST to platelet index; FIB-4: Fibrosis-4 index; HSI: hepatic steatosis index.

**Table 2:** Odds ratios for NAFLD according to tertiles of each parameter.

Parameter	Tertile (Range)	OR (95% Cl)	*p*
TC/HDL-C			
	T1 (1.78, 2.80)	Reference	
	T2 (2.80, 3.45)	3.721 (0.750–18.448)	0.108
	T3 (3.45, 5.80)	12.724 (2.871–56.388)	<0.001
TG/HDL-C			
	T1 (0.43, 1.21)	Reference	
	T2 (1.21, 1.81)	3.149 (0.618–16.061)	0.168
	T3 (1.81, 21.14)	13.566 (3.070–59.951)	<0.001
LDL-C/HDL-C			
	T1 (0.56, 1.62)	Reference	
	T2 (1.62, 2.14)	3.115 (0.610–15.899)	0.172
	T3 (2.15, 3.82)	13.715 (3.099–60.701)	<0.001
Non-HDL-C/HDL-C			
	T1 (0.78, 1.80)	Reference	
	T2 (1.80, 2.45)	3.721 (0.750–18.448)	0.108
	T3 (2.45, 4.80)	12.724 (2.871–56.388)	<0.001
TyG			
	T1 (6.94, 8.03)	Reference	
	T2 (8.04, 8.38)	2.049 (0.496–8.473)	0.322
	T3 (8.39, 10.28)	8.513 (2.424–29.896)	<0.001
TyG-BMI			
	T1 (111.76, 161.92)	Reference	
	T2 (161.99, 191.35)	3.035 (0.120–76.973)	0.501
	T3 (191.55, 442.29)	84.284 (4.964–1431.064)	0.002
TyG-BMI SDS			
	T1 (−36.73, −5.27)	Reference	
	T2 (−5.27, 3.40)	3.035 (0.120–76.973)	0.501
	T3 (3.64, 73.23)	84.284 (4.964–1431.064)	0.002
TyG-WC			
	T1 (433.82, 563.56)	Reference	
	T2 (563.71, 616.19)	0.974 (0.018–53.130)	0.990
	T3 (618.63, 1257.24)	27.869 (1.504–516.395)	0.026
TyG-WHR			
	T1 (4.81, 5.94)	Reference	
	T2 (5.94, 6.57)	7.271 (0.363–145.773)	0.195
	T3 (6.61, 10.76)	73.974 (4.334–1262.656)	0.003
TyG-WHtR			
	T1 (2.55, 3.32)	Reference	
	T2 (3.32, 3.67)	0.974 (0.018–53.130)	0.990
	T3 (3.69, 6.95)	27.869 (1.504–516.395)	0.026
APRI			
	T1 (0.15, 0.25)	Reference	
	T2 (0.26, 0.33)	1.319 (0.468–3.719)	0.600
	T3 (0.33, 1.60)	2.010 (0.760–5.314)	0.159
APRI-BMI			
	T1 (2.64, 5.46)	Reference	
	T2 (5.47, 7.23)	3.721 (0.750–18.448)	0.108
	T3 (7.26, 27.63)	12.724 (2.871–56.388)	<0.001
APRI-BMI SDS			
	T1 (−3.42, −0.20)	Reference	
	T2 (−0.19, 0.13)	3.035 (0.120–76.973)	0.501
	T3 (0.14, 2.22)	84.284 (4.964–1431.064)	0.002
APRI-WC			
	T1 (13.08, 20.35)	Reference	
	T2 (20.77, 25.77)	3.088 (0.306–31.170)	0.339
	T3 (25.83, 74.34)	6.774 (0.772–59.418)	0.084
APRI-WHR			
	T1 (0.10, 0.19)	Reference	
	T2 (0.19, 0.26)	0.789 (0.204–3.054)	0.732
	T3 (0.26, 0.78)	4.672 (1.649–13.238)	0.004
APRI-WHtR			
	T1 (0.08, 0.12)	Reference	
	T2 (0.13, 0.15)	1.500 (0.236–9.552)	0.668
	T3 (0.15, 0.42)	2.656 (0.481–14.677)	0.263
FIB-4			
	T1 (1.82, 3.76)	Reference	
	T2 (3.79, 5.84)	2.451 (0.612–9.814)	0.205
	T3 (5.85, 45.91)	7.846 (2.227–27.645)	0.001
HSI			
	T1 (17.88, 26.82)	Reference	
	T2 (26.90, 30.80)	5.000 (0.232–107.601)	0.304
	T3 (30.86, 65.70)	79.035 (4.650–1343.230)	0.003

ORs and 95% CIs of tertiles 2–3 for each parameter were calculated and compared with those of tertile 1 as a reference. NAFLD: non-alcoholic fatty liver disease; TC: total cholesterol; TG: triglycerides; AST: aspartate aminotransferase; ALT: alanine aminotransferase; HDL-C: high-density lipoprotein cholesterol; LDL-C: low-density lipoprotein cholesterol; TyG: triglyceride glucose index; BMI: body mass index; SDS: standard deviation score; WC: waist circumference; WHR: waist-to-hip ratio; WHtR: waist-to-height ratio; APRI: AST to platelet index; FIB-4: Fibrosis-4 index; HSI: hepatic steatosis index.

**Table 3:** Cut-off values and areas under the receiver operating characteristics curves for each parameter for predicting insulin resistance.

Parameter	Cut-Off	Sensitivity	Specificity	NPV	AUC	95% CI	*p*
TyG	8.466	65.517	80.349	94.845	0.761	(0.658–0.864)	<0.001
TyG-BMI	201.617	89.655	87.773	98.529	0.941	(0.908–0.974)	<0.001
TyG-BMI SDS	3.809	96.552	76.419	99.432	0.924	(0.887–0.960)	<0.001
TyG-WC	682.997	100.000	93.000	100	0.972	(0.943–1.000)	<0.001
TyG-WHR	6.708	89.286	79.717	98.256	0.923	(0.877–0.970)	<0.001
TyG-WHtR	3.955	90.000	89.000	98.889	0.947	(0.896–0.998)	<0.001
APRI	0.458	24.138	90.393	90.393	0.575	(0.456–0.693)	0.217
APRI-BMI	7.923	65.517	76.856	94.624	0.773	(0.685–0.86)	<0.001
APRI-BMI SDS	0.283	89.655	85.153	98.485	0.926	(0.89–0.962)	<0.001
APRI-WC	23.756	80.000	55.000	96.491	0.684	(0.53–0.838)	0.019
APRI-WHR	0.275	64.286	77.830	94.286	0.708	(0.601–0.815)	<0.001
APRI-WHtR	0.143	80.000	57.000	96.610	0.662	(0.495–0.829)	0.057
FIB-4	5.201	75.862	63.319	95.395	0.737	(0.635–0.838)	<0.001
HSI	31.680	93.103	82.969	98.958	0.929	(0.889–0.968)	<0.001

NPV: negative predictive value; AUC: area under the curve; CI: confidence interval; NAFLD: non-alcoholic fatty liver disease; TyG: triglyceride glucose index; BMI: body mass index; SDS: standard deviation score; WC: waist circumference; WHR: waist-to-hip ratio; WHtR: waist-to-height ratio; APRI: aspartate aminotransferase to platelet index; FIB-4: Fibrosis-4 index; HSI: hepatic steatosis index.

## Data Availability

The data that support the findings of this study are available from the corresponding author upon reasonable request. The data are not publicly available due to privacy rights of the participants.

## Supplementary Materials

The following supporting information can be downloaded at: https://www.mdpi.com/article/10.3390/biology11050685/s1, Table S1: Supplemental characteristics of the participants according to NAFLD; Table S2: Comparison of areas under receiver operating curves among each parameter for predicting NAFLD; Figure S1: APRI, modified APRIs, FIB-4, and HSI according to the NAFLD grade.
