# Supplementary material for: Comparison of the Modified TyG Indices and Other Parameters to Predict Non-Alcoholic Fatty Liver Disease in Youth
Source: Biology (Basel). 2022 Apr 29;11(5):685. doi: 10.3390/biology11050685 (PMC9138077; doi:10.3390/biology11050685)
Supplement: Supplementary file 1 [file biology-11-00685-s001.zip › biology-1681292-supplementary.pdf]

SUPPLEMENTARY DATA

Comparison of the Modified TyG Indices and Other Parameters to Predict Non-Alcoholic Fatty Liver Disease in Youth

Kyungchul Song [1], Hae Won Lee [1], Han Saem Choi [2], Goeun Park [3], Hye Sun Lee [3], Su Jin Kim [1], Myeongseob Lee [1], Junghwan Suh [1], Ahreum Kwon [1], Ho-Seong Kim [1] and Hyun Wook Chae [1,*]

[1]Department of Pediatrics, Severance Children's Hospital, Endocrine Research Institute, Yonsei University College of Medicine, Seoul, Korea
[2]Department of Pediatrics, Catholic Kwandong University, International St. Mary's Hospital, Incheon, South Korea
[3]Biostatistics Collaboration Unit, Yonsei University College of Medicine, Seoul, Korea

Corresponding author: Hyun Wook Chae
Department of Pediatrics, Severance Children's Hospital, Endocrine Research Institute, Yonsei University College of Medicine, 50-1 Yonsei-ro, Seodaemun-gu, Seoul 03722, Korea; E-mail: hopechae@yuhs.ac, Tel: +82-2-2019-3350, Fax: +82-2-393-9118

Table S1. Supplemental characteristics of the participants according to NAFLD

Characteristics	Total (n = 258)	Normal (n = 229)	NAFLD (n = 29)	p
Height, cm	168.66 ± 8.44	168.06 ± 8.27	173.4 ± 8.42	0.001
Weight, kg	63.1 ± 15.01	60.25 ± 11.46	85.56 ± 20.27	< 0.001
Platelet, ×10^3/μL	272.23 ± 47.47	271.35 ± 47.83	279.17 ± 44.63	0.404
Albumin, g/dL	4.8 ± 0.25	4.79 ± 0.25	4.84 ± 0.26	0.351
Total Bilirubin, mg/dL	0.9 ± 0.41	0.9 ± 0.42	0.84 ± 0.36	0.459
ALP, IU/L	82.22 ± 32.2	80.97 ± 31.45	92.03 ± 36.8	0.081
γ-GT, IU/L	17.09 ± 10.26	15.44 ± 7.09	30.14 ± 18.9	< 0.001
TC, mg/dL	167.43 ± 27.13	166.3 ± 27.44	176.31 ± 23.1	0.061
TG, mg/dL	92.16 ± 55.36	86.01 ± 47.73	140.69 ± 82.61	0.002
HDL-C, mg/dL	54.54 ± 11.22	55.61 ± 10.95	46.07 ± 9.85	< 0.001
LDL-C, mg/dL	94.44 ± 24.45	93.48 ± 24.86	102.03 ± 19.74	0.076
Non-HDL-C, mg/dL	112.89 ± 25.87	110.69 ± 25.34	130.24 ± 23.71	< 0.001

Continuous variables are presented mean ± standard deviation and categorical variables as number (percentages). p value is assessed using independent t-test for continuous variables and Chi-square test for categorical variables. NAFLD: Non-alcoholic fatty liver disease; ALP: alkaline phosphatase; γ-GT: Gamma-glutamyl transferase; TC: total cholesterol; TG: triglycerides; HDL-C: high-density lipoprotein cholesterol; LDL-C: low-density lipoprotein cholesterol.

Figure S1. APRI, modified APRIs, FIB-4, and HSI according to the NAFLD grade

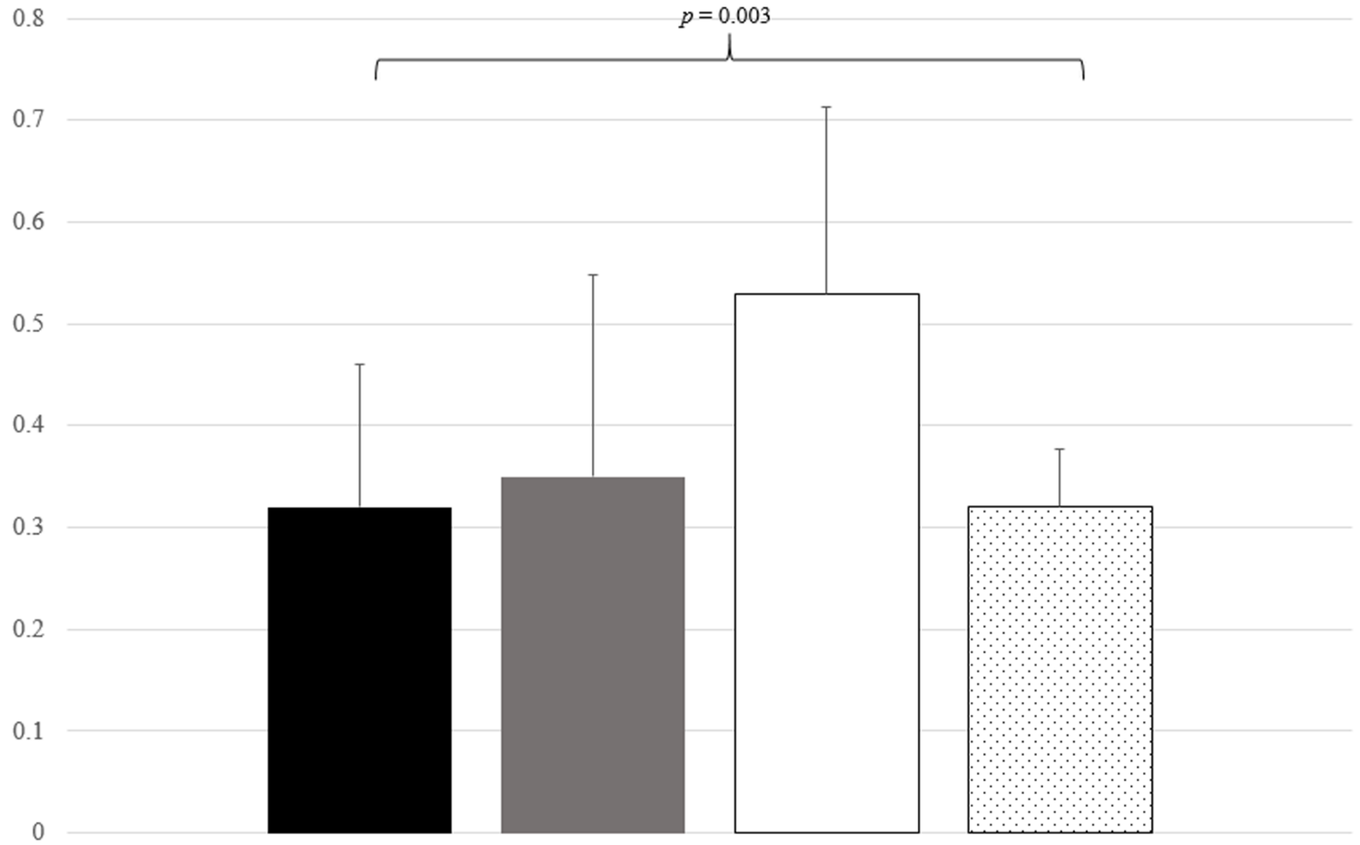

(a)

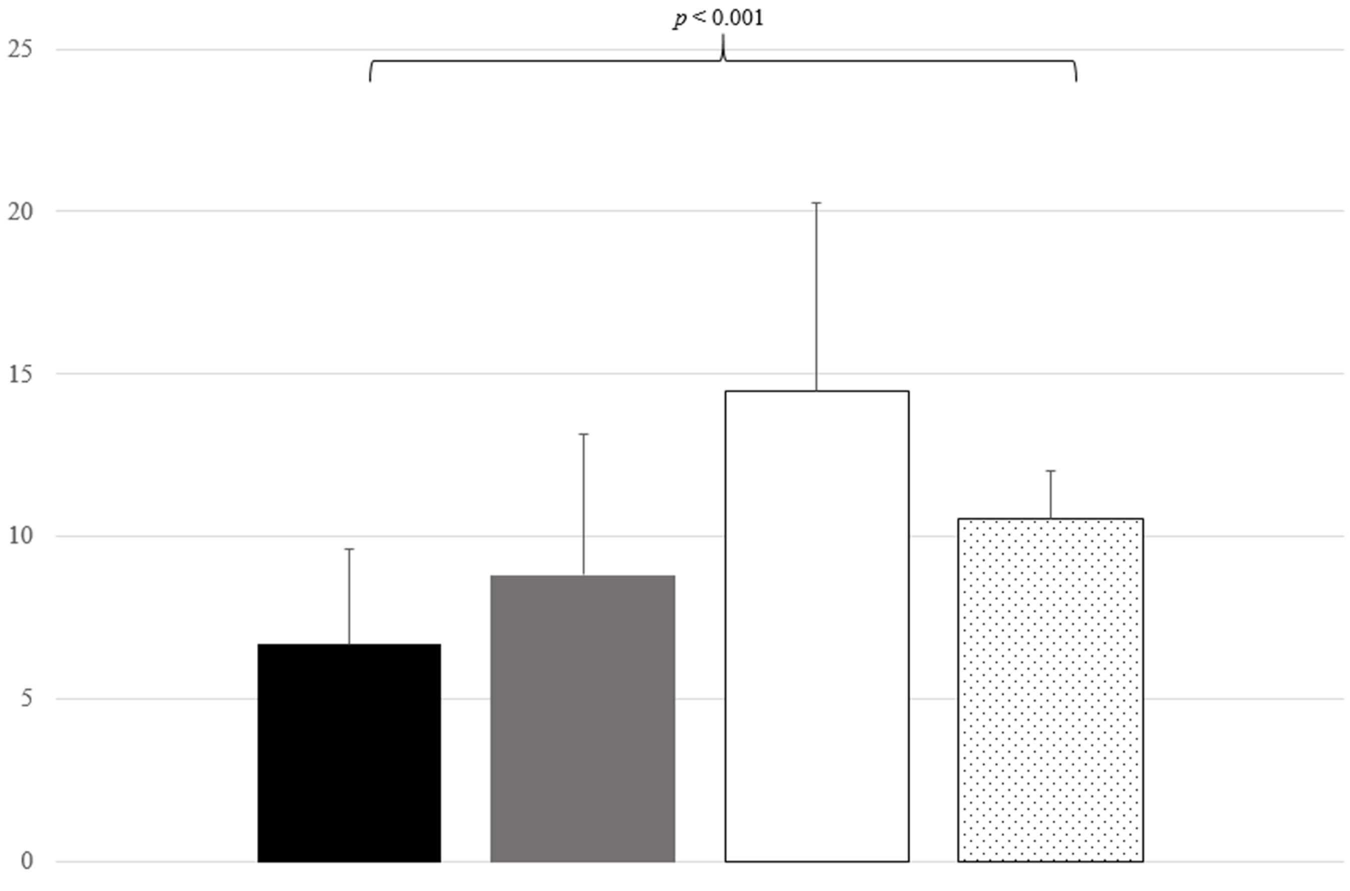

(b)

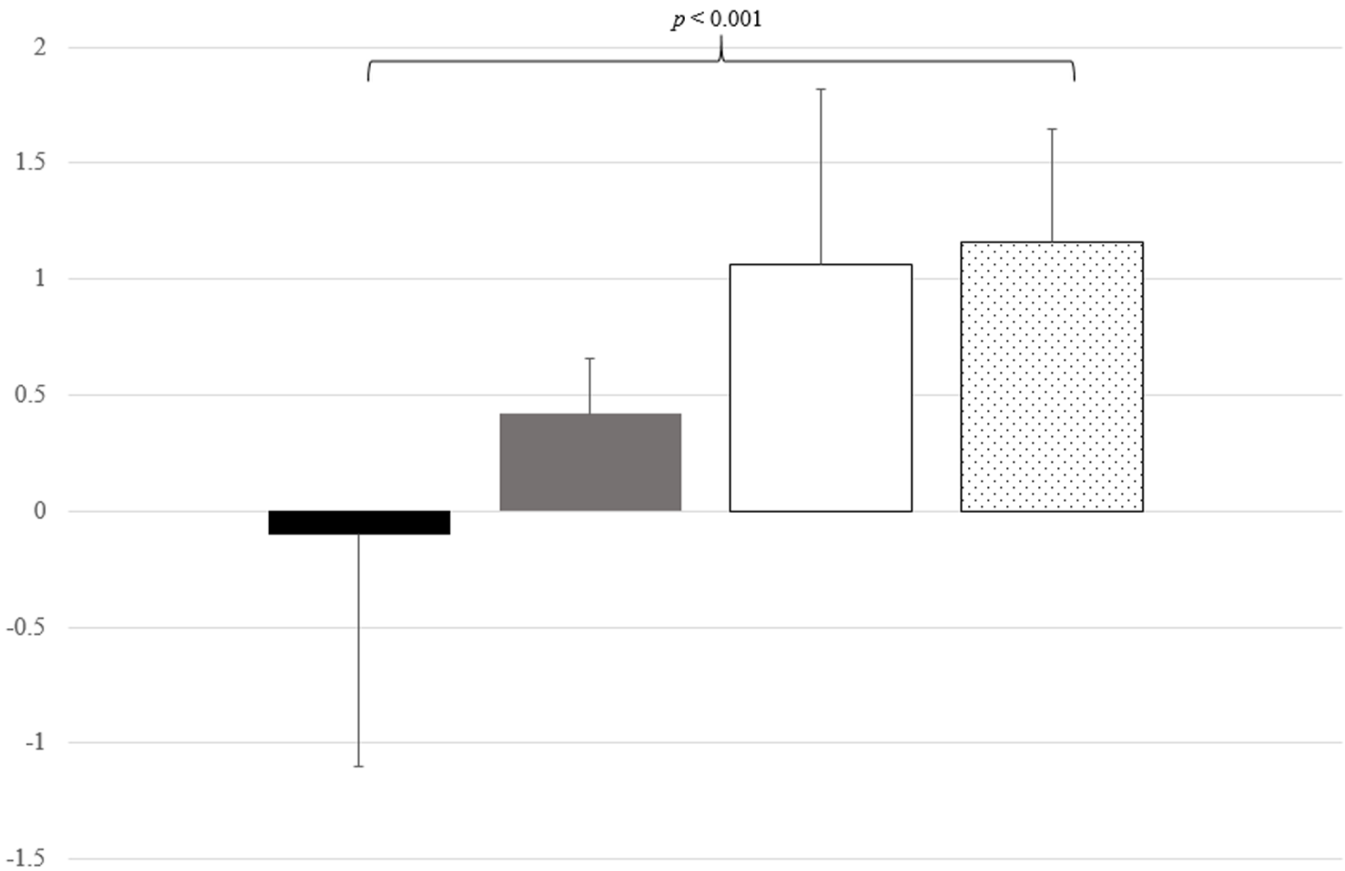

(c)

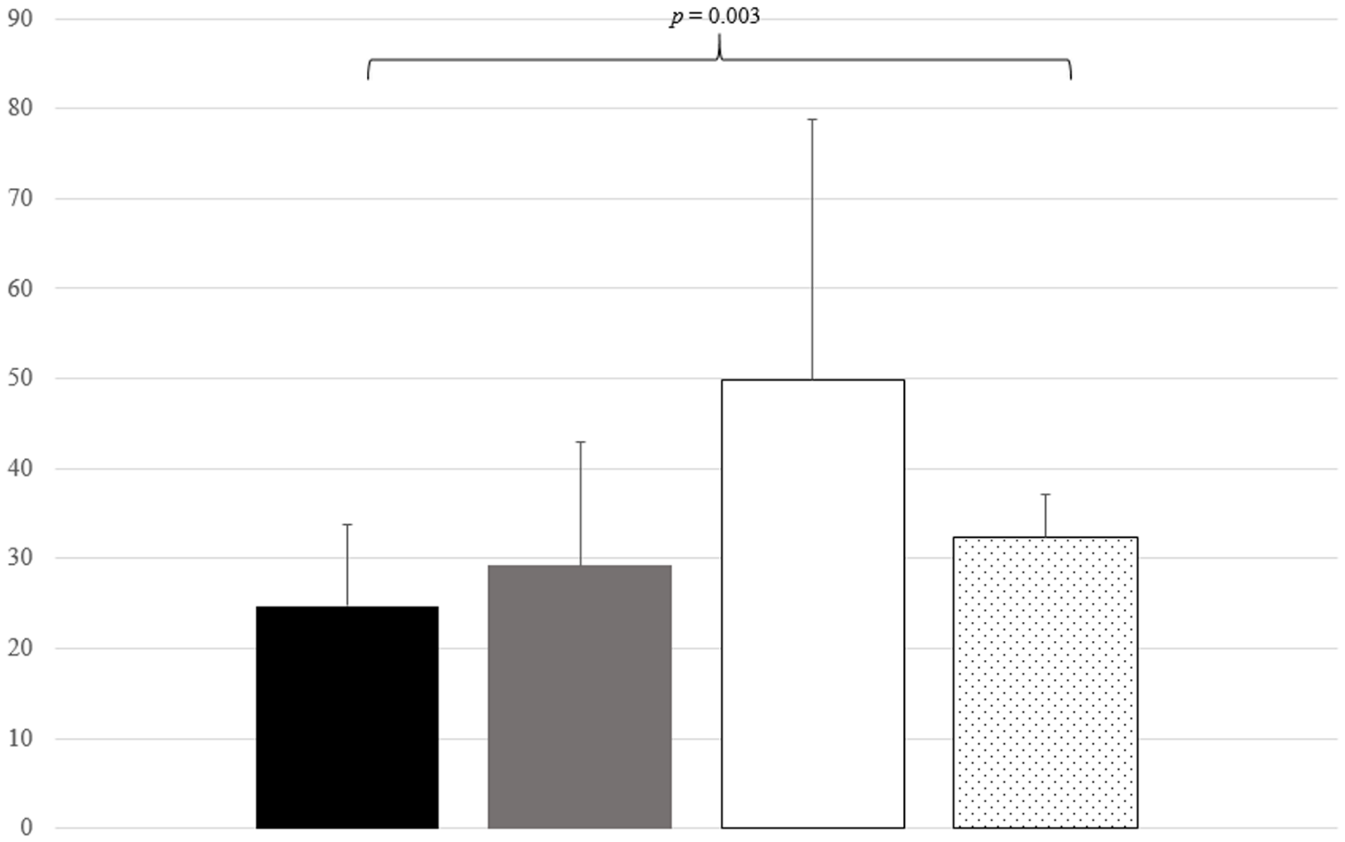

(d)

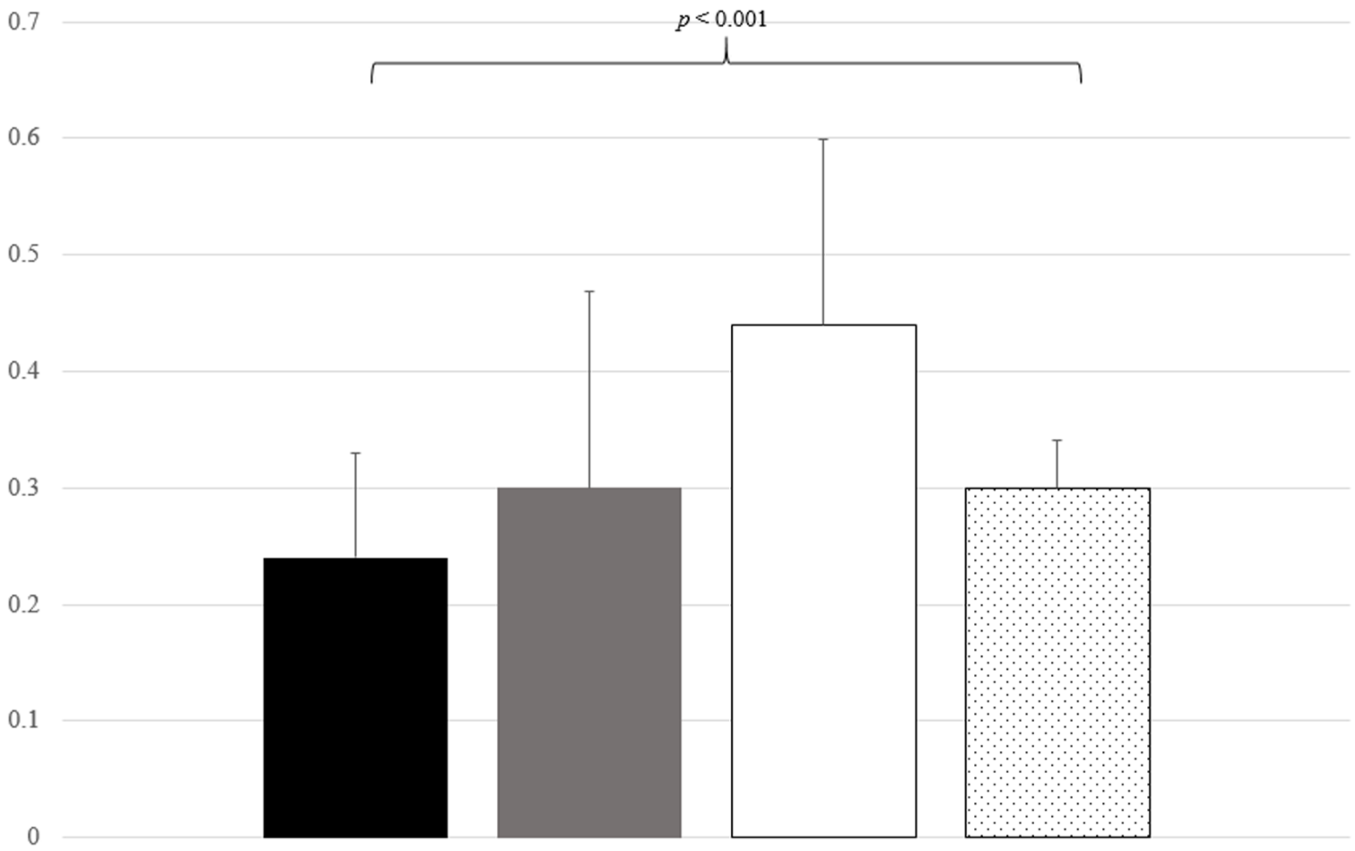

p < 0.001
0.7
0.6
0.5
0.4
0.3
0.2
0.1
0

(e)

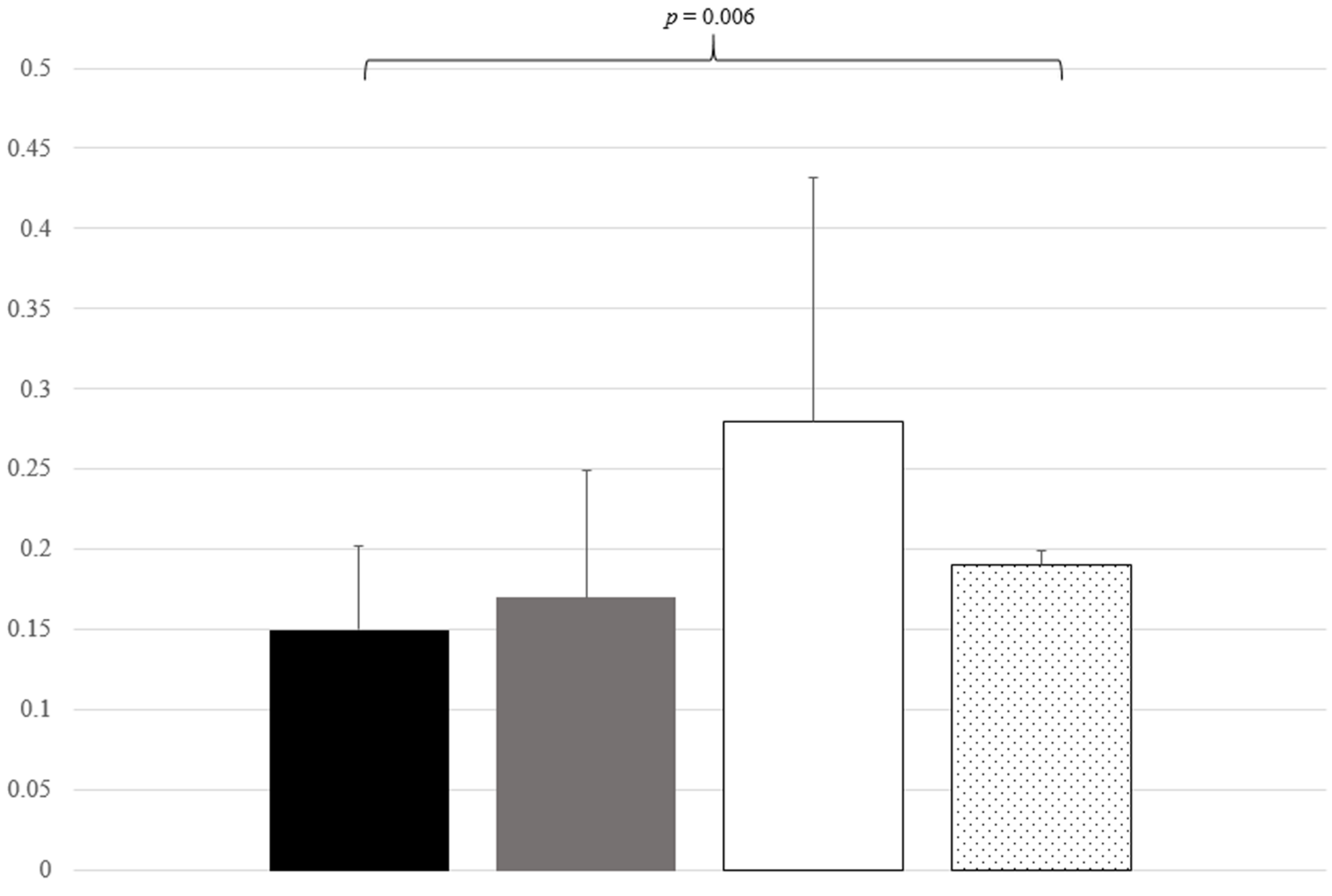

p = 0.006
0.5
0.45
0.4
0.35
0.3
0.25
0.2
0.15
0.1
0.05
0

(f)

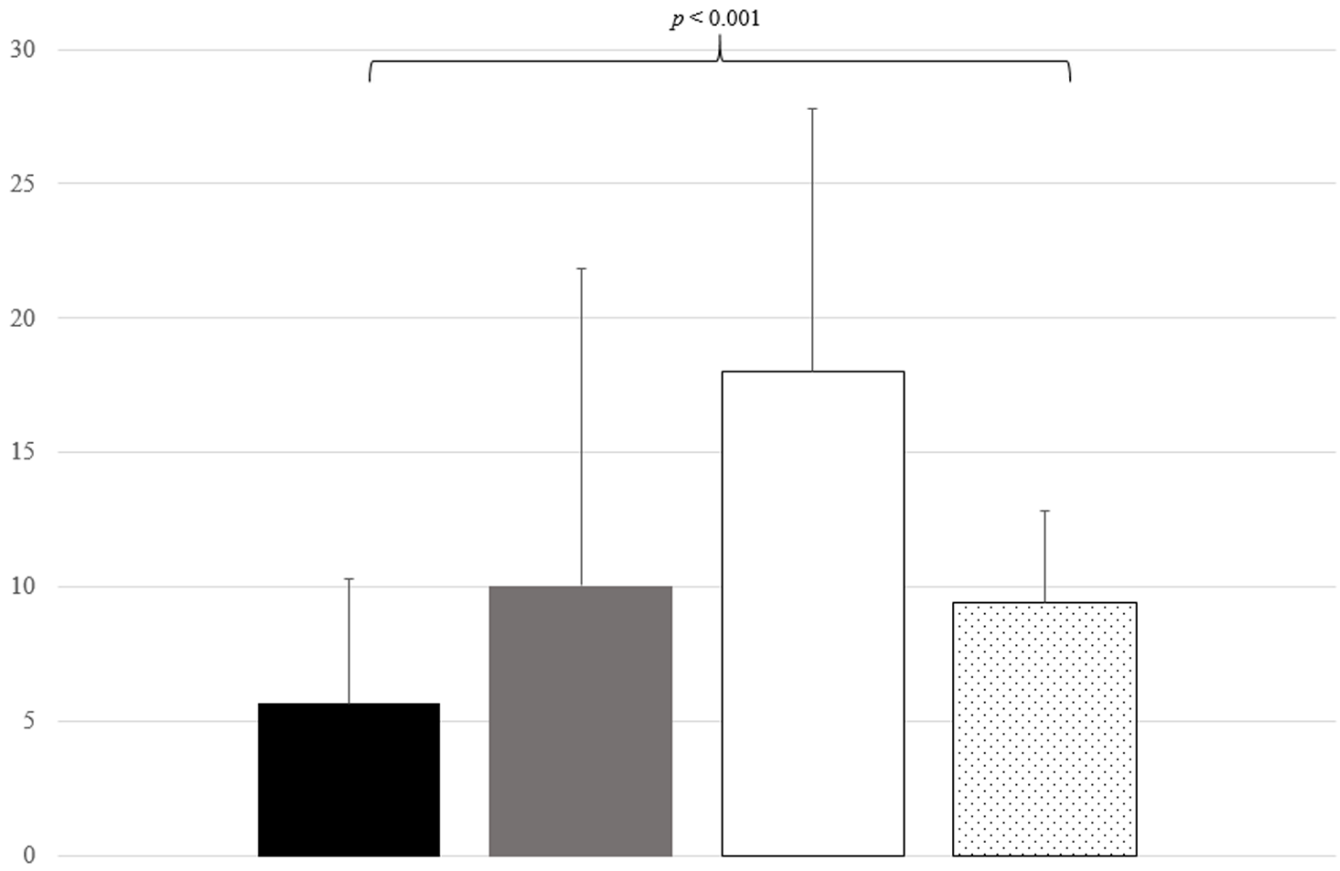

(g)

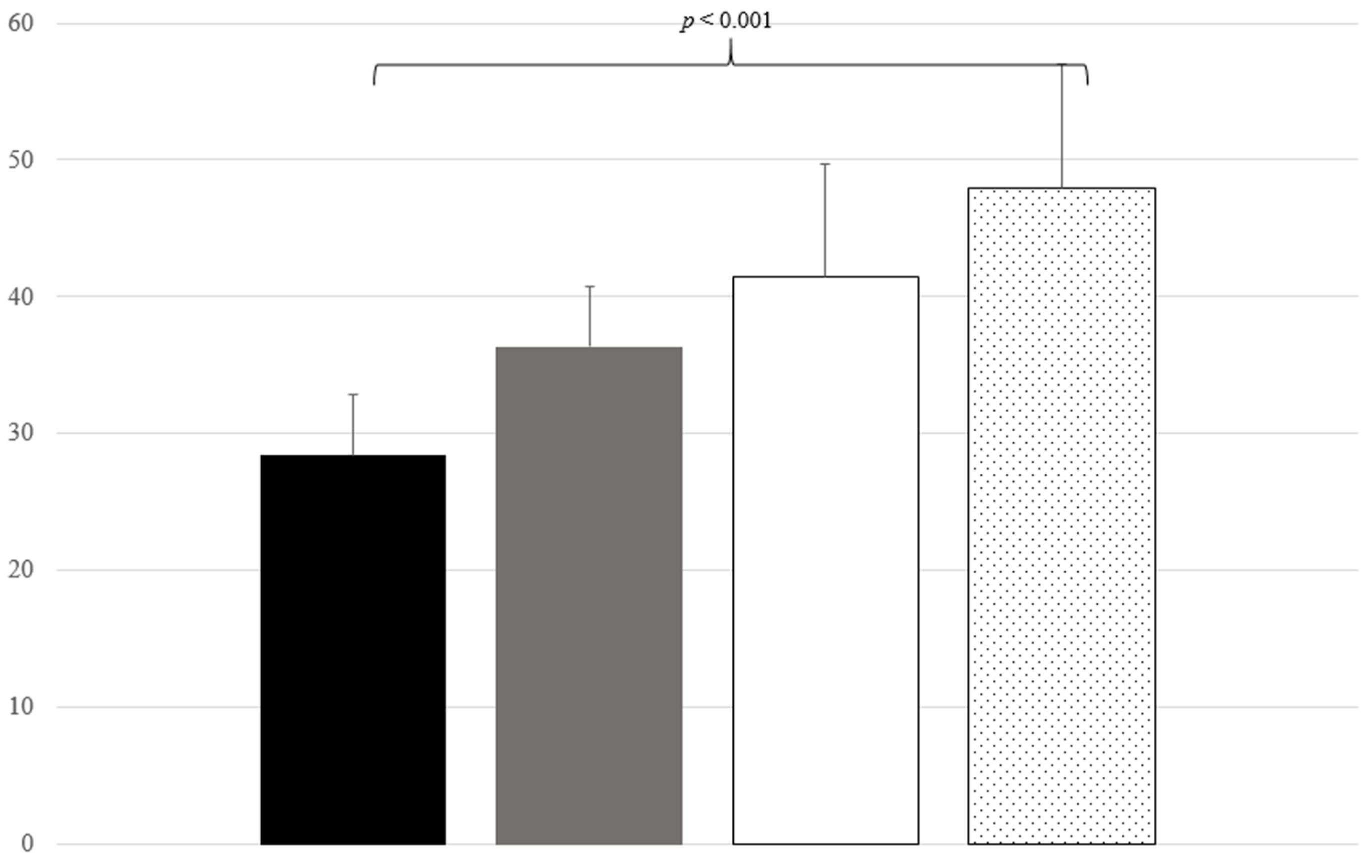

(h)

The black bar is the NAFLD grade group, the gray bar is the NAFLD grade 1 group, the white bar is the NAFLD grade 2 group, and the bar with dot pattern is the NAFLD grade 3 group. The number on the bar is the p-value of analysis of variance. **(a)** APRI index according to the NAFLD grade; **(b)** APRI-BMI according to the NAFLD grade; **(c)** APRI-BMI SDS according to the NAFLD grade; **(d)** APRI-WC according to the NAFLD grade; **(e)** APRI-WHR according to the NAFLD grade; **(f)** APRI-WHtR according to the NAFLD grade; **(g)** FIB-4 according to the NAFLD grade; **(h)** HSI according to the NAFLD grade; APRI: aspartate aminotransferase to platelet index; FIB-4: fibrosis-4 index; HSI: hepatic steatosis index; NAFLD: non-alcoholic fatty liver disease; BMI: body mass index; SDS: standard deviation score; WC: waist circumference; WHR: waist-to-hip ratio; WHtR: waist-to-height ratio.

Table S2. Comparison of areas under receiver operating curves among each parameter for predicting NAFLD

Parameter	TyG	TyG-BMI	TyG-BMI SDS	TyG-WC	TyG-WHR	TyG-WHtR	APRI	APRI-BMI	APRI-BMI SDS	APRI-WC	APRI-WHR	APRI-WHtR	FIB-4	HSI
TyG	reference	< 0.001	0.003	< 0.001	< 0.001	< 0.001	0.008	0.833	0.002	0.391	0.442	0.298	0.676	< 0.001
TyG-BMI	< 0.001	reference	0.157	0.068	0.261	0.824	< 0.001	< 0.001	0.173	0.001	< 0.001	0.002	< 0.001	0.359
TyG-BMI SDS	0.003	0.157	reference	0.007	>0.999	0.337	< 0.001	< 0.001	0.824	0.002	< 0.001	< 0.001	< 0.001	0.739
TyG-WC	< 0.001	0.068	0.007	reference	0.041	0.141	< 0.001	< 0.001	0.011	< 0.001	< 0.001	< 0.001	< 0.001	0.056
TyG-WHR	< 0.001	0.261	>0.999	0.041	reference	0.439	< 0.001	< 0.001	0.886	0.003	< 0.001	0.003	< 0.001	0.726
TyG-WHtR	< 0.001	0.824	0.337	0.141	0.439	reference	< 0.001	< 0.001	0.379	< 0.001	< 0.001	< 0.001	< 0.001	0.549
APRI	0.008	< 0.001	< 0.001	< 0.001	< 0.001	< 0.001	Reference	< 0.001	< 0.001	0.178	< 0.001	0.317	< 0.001	< 0.001
APRI-BMI	0.833	< 0.001	< 0.001	< 0.001	< 0.001	< 0.001	< 0.001	reference	< 0.001	0.216	0.004	0.162	0.174	< 0.001
APRI-BMI SDS	0.002	0.173	0.824	0.011	0.886	0.379	< 0.001	< 0.001	reference	0.001	< 0.001	0.001	< 0.001	0.814
APRI-WC	0.391	< 0.0001	0.002	< 0.001	0.003	< 0.001	0.178	0.216	0.001	reference	0.739	0.317	0.518	0.002
APRI-WHR	0.442	< 0.001	< 0.001	< 0.001	< 0.001	< 0.001	< 0.001	0.004	< 0.001	0.739	reference	0.562	0.378	< 0.001
APRI-WHtR	0.298	0.002	< 0.001	< 0.001	0.003	< 0.001	0.317	0.162	0.001	0.317	0.562	reference	0.403	0.002
FIB-4	0.676	< 0.001	< 0.001	< 0.001	< 0.001	< 0.001	< 0.001	0.174	< 0.001	0.518	0.378	0.403	reference	< 0.001
HSI	< 0.001	0.359	0.739	0.056	0.726	0.549	< 0.001	< 0.001	0.814	0.002	< 0.001	0.002	< 0.001	reference

Note: Bootstrap method was used to perform pairwise comparisons between AUCs for the parameters. Values are presented as p values. NAFLD: Non-alcoholic fatty liver disease; TyG: triglyceride glucose index; BMI: body mass index; SDS: standard deviation score; WC: waist circumference; WHR: waist-to-hip ratio; WHtR: waist-to-height ratio; APRI: aspartate aminotransferase to Platelet index; FIB: Fibrosis-4 index; HSI: hepatic steatosis index.
